# Protection against MIS-C outweighs the risk of myocarditis after Covid-19 vaccination in children

**DOI:** 10.1186/s13052-022-01335-1

**Published:** 2022-08-04

**Authors:** Marcello Mariani, Roberta Caorsi, Alessandro Consolaro, Giacomo Brisca, Camilla Sticchi, Marco Gattorno, Elio Castagnola, Angelo Ravelli

**Affiliations:** 1 Infectious Diseases Unit and COVID Hospital, IRCCS Istituto Giannina Gaslini, Genoa, Italy; 2 Department of Neuroscience, Rehabilitation, Ophthalmology, Genetics, Maternal and Child Health (DINOGMI), University of Genova, Genoa, Italy; 3 Clinic of Paediatrics and Rheumatology, IRCCS Istituto Giannina Gaslini, Genoa, Italy; 4 Subintensive Care Unit COVID-19, IRCCS Istituto Giannina Gaslini, Genoa, Italy; 5 Regional Health Agency of Liguria (ALiSa), Genoa, Italy; 6 Scientific Directory, IRCCS Istituto Giannina Gaslini, Genoa, Italy

**Keywords:** Vaccine, BNT162b2, Children

## Abstract

From March 2020 to July 2022, in Liguria region (North-West Italy) incidence of MIS-C among pediatric patients infected by SARS-CoV-2 was 38.7/100.000, which is higher than that of myocarditis after COVID-19 vaccination. In our opinion severity of MIS-C-related cardiac disease outweigh the risk of myocarditis after COVID-19 vaccine.

## Main text

To the editor.

A number of studies have reported an increased rate of myocarditis among subjects who received mRNA vaccination against COVID-19. The highest incidence was seen in young male adolescents and adults (16 to 29 years of age), with 8.62 excess events per 100,000 persons (95% confidence interval, 2.82 to 14.35 [1]. Symptoms of myocarditis developed most frequently within a few days after the second dose of vaccine [2, 3]. However, the clinical presentation was generally mild, with resolution of myocarditis in most cases and a relatively short length of hospital stay [3]. Although the mechanism of vaccine-induced myocarditis is not known, it may be related to the active component of the vaccine, the mRNA sequence that codes for the spike protein of severe acute respiratory syndrome coronavirus 2 (SARS-CoV-2), or to the immune response that follows vaccination [3].

The risk of myocarditis is one of the reasons for the mistrust regarding the safety of COVID-19 vaccines in children. However, on June 23rd 2021, the Center for Disease Control (CDC)‘s Advisory Committee on Immunization Practices (ACIP) reviewed the available data and concluded that the benefits of COVID-19 vaccination to the individual persons and the population outweighs the risk of myocarditis and recommended the continued use of the vaccine in subjects aged ≥12 years [2]. In a multinational, placebo-controlled trial of the BNT162b2 COVID-19 vaccine in adolescents 12 to 15 years of age, the vaccine was found to have a favorable safety and side-effect profile, with mainly transient mild-to-moderate reactogenicity (predominantly injection-site pain, fatigue, and headache). Through an up to 1-month follow-up after the second dose, no case with myocarditis was observed [4]. In a subsequent randomized trial, 1517 children aged 5 to 11 years were given two doses of the BNT162b2 vaccine administered 21 days apart. After a median follow-up of 2.3 months, no vaccine-related adverse events, including myocarditis, were noted [5].

One of the main benefits of preventing SARS-CoV-2 infection in children and adolescents include the protection against the multisystem inflammatory syndrome in children (MIS-C), which is the most serious and worrying complication of COVID-19 in the pediatric age group [6]. This condition is thought to be caused by a post-infectious inflammatory process and manifests clinically with signs and symptom similar to those of Kawasaki disease (KD), but is also marked by clinical manifestations unusual in KD, particularly gastrointestinal complaints and myocarditis, often leading to myocardial failure and shock [7]. The severity of cardiac involvement often requires admission to the intensive care unit, a long hospital stay, and an aggressive therapeutic approach [8, 9]. Thus far, the risk of long-term sequelae to the heart is unknown but is being investigated. As of November 1, 2021, a total of 5526 patients meeting case definition for MIS-C have been reported to the CDC, 48 of whom had died [10].

Istituto Giannina Gaslini is a third-level pediatric hospital acting as referral center for SARS COV2-related diseases in Liguria Region (North-West Italy). From March 2020 to July 2022, MIS-C was diagnosed in 37 patients with a median age of 6 years (25th-75th centile: 3-11 years).

Clinical and population characteristics are resumed in Table 1: more than 97% of MIS-C patients had fever > 38 °C; rash and fatigue were present in 62% of cases. Other common symptoms were abdominal pain and nausea or vomiting, recorded in more than 50% of cases.

**Table 1:** Clinical characteristics of MIS-C population hospitalized between March 2020 and July 2022 at Istituto Giannina Gaslini

	0-11 months		1-5 years		6-10 years		11-18 years		total	
Sex	**n**	**% on total**	**n**	**% on total**	**n**	**% on total**	**n**	**% on total**	**n**	**%**
Male	1	2,7	8	21,6	6	16,2	8	21,6	23	62,2
Female	0	0,0	8	21,6	3	8,1	3	8,1	14	37,8
Total	1		16		9		11			
Hospital stay	**n**	**25-75 centile**	**n**	**25-75 centile**	**n**	**25-75 centile**	**n**	**25-75 centile**		
Days	20		13,5	(11-19,5)	17	(15-19)	20	(16,5-23,5)		
Symptoms upon hospitalization	**n**	**% on same age group**	**n**	**% on same age group**	**n**	**% on same age group**	**n**	**% on same age group**	**n**	**%**
Fever > 38 °C	1	100	15	93,8	9	100	11	100	36	97,3
Cough	0	0	1	6,3	1	11,1	2	18,2	4	10,8
Fatigue	1	100	9	56,3	7	77,8	6	54,5	23	62,2
Rhinitis	1	100	16	100,0	9	100	11	100	37	100,0
Nausea/vomiting	1	100	9	56,3	5	55,6	6	54,5	21	56,8
Abdominal pain	1	100	7	43,8	6	66,7	7	63,6	21	56,8
Hypotension	0	0	0	0,0	4	44,4	4	36,4	8	21,6
Respiratory distress	0	0	0	0,0	0	0	1	9,1	1	2,7
Feeding difficulties	0	0	7	43,8	4	44,4	4	36,4	15	40,5
Rash	1	100	12	75,0	7	77,8	3	27,3	23	62,2
Myalgia	0	0	2	12,5	2	22,2	1	9,1	5	13,5
Laboratory tests upon arrival	**median**	**25th-75th centile**	**median**	**25th-75th centile**	**median**	**25th-75th centile**	**median**	**25th-75th centile**		
Leucocytes count/mmc	10,540		10,275	6683-11,805	10,690	6510-14,390	8400	5645-13,185		
Lymphocytes count/mmc	3860		1945	1443-3280	1170	890-1640	680	400-1500		
CRP (mg/dl)	15,1		9,62	6,87-16,2	7,1	4,24-13,4	19,4	16,1-22,2		
D-dimer (ug/ml)	//		2,45	1,62-4,65	4,73	2,73-6,34	2,31	1,67-2,88		
NT-pro BNP (pg/ml)	//		1479	1056-2684	1524	833-2024	806	266-7311		
Therapy	**n**	**% on same age group**	**n**	**% on same age group**	**n**	**% on same age group**	**n**	**% on same age group**		
Immunotherapy (steroids, immunoglobulin, anakinra)	1	100	15	93,8	9	100	11	100		
Anticoagulants	1	100	15	93,8	8	88,9	10	90,9		

No patient died, but all needed hospitalization for more than 10 days in children of 1-5 years and of more than 15 days in other ages. Immunotherapy (intended as immunoglobulins, steroids or anakinra) was administered in more than 90% of patients as well as anticoagulants.

**Fig. 1:**
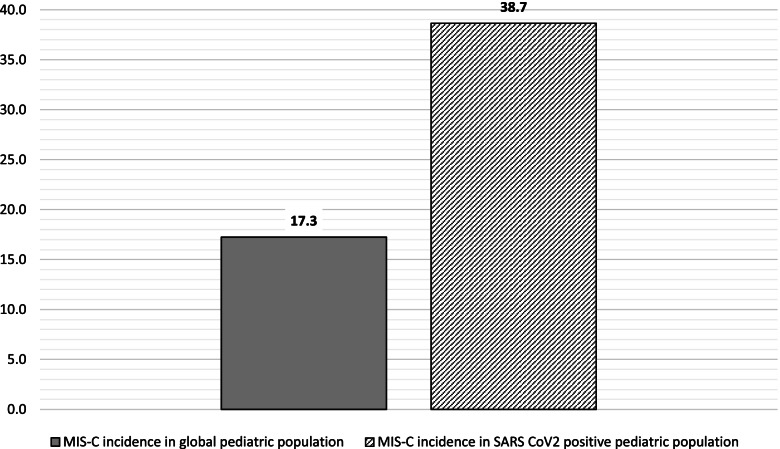
Incidence comparison between MIS-C indicence in pediatric population from Liguria Region, Italy in 2020-2022. Data are expressed as cases * 100.000 inhabitants under 19 years of age

Meanwhile, a total of 95.693 subjects aged < 19 years were diagnosed with COVID-19 in Liguria, making an incidence of MIS-C among pediatric patients infected by SARS-CoV-2 of 38.7/100.000, which is higher than that of myocarditis after COVID-19 vaccination (Fig. 1).

The incidence of MIS-C on COVID-19 positive pediatric population is therefore about two times higher compared to incidence we already documented on global regional pediatric population [11].

In our view, the higher incidence of MIS-C after COVID-19 infection and the much greater severity of MIS-C-related cardiac disease outweigh the risk of myocarditis after COVID-19 vaccine and their prevention represent important reasons for performing the vaccination in children and adolescents.

## Data Availability

Datasets used and/or analysed during the current study are available from the corresponding author on reasonable request.

## Abbreviations

COVID-19: Corona Virus Disease 19
MIS-C: multisystem inflammatory syndrome in children
